# Testing Alcohol Labels as a Tool to Communicate Cancer Risk to Drinkers: A Real-World Quasi-Experimental Study

**DOI:** 10.15288/jsad.2020.81.249

**Published:** 2020-05-03

**Authors:** Erin Hobin, Ashini Weerasinghe, Kate Vallance, David Hammond, Jonathan McGavock, Thomas K. Greenfield, Nour Schoueri-Mychasiw, Catherine Paradis, Tim Stockwell

**Affiliations:** ^a^ Public Health Ontario, Toronto, Ontario, Canada; ^b^ Dalla Lana School of Public Health, University of Toronto, Toronto, Ontario, Canada; ^c^ Canadian Institute for Substance Use Research, University of Victoria, Victoria, British Columbia, Canada; ^d^ School of Public Health and Health Systems, University of Waterloo, Waterloo, Ontario, Canada; ^e^ Children’s Hospital Research Institute of Manitoba, University of Manitoba, Winnipeg, Manitoba, Canada; ^f^ Alcohol Research Group, Public Health Institute, Emeryville, California; ^g^ Canadian Centre for Substance Use and Addiction, Ottawa, Ontario, Canada

## Abstract

**Objective:**

This study tested the initial and continued effects of cancer warning labels on drinkers’ recall and knowledge that alcohol can cause cancer.

**Method:**

A quasi-experiment was conducted to examine changes in the intervention versus comparison site for three outcomes: unprompted and prompted recall of the cancer warning, and knowledge that alcohol can cause cancer. The intervention site applied cancer warning labels to alcohol containers in its liquor store for 1 month, and the two liquor stores in the comparison site did not apply cancer labels. In total, 2,049 unique cohort participants (1,056 male) were recruited at liquor stores in the intervention and comparison sites to participate in surveys 4 months before labels were applied and 2 and 6 months after the cancer label was halted because of alcohol industry interference. Generalized estimating equations tested differences in outcomes between sites over time adjusting for socio-demographics and other covariates.

**Results:**

Two months after the cancer label, unprompted (+24.2% vs. +0.6%; adjusted odds ratio [AOR] = 32.7, 95% CI [5.4, 197.7]) and prompted (+35.7% vs. +4.1%; AOR = 6.2, 95% CI [3.6, 10.9]) recall increased to a greater extent in the intervention versus comparison site. There was a 10% greater increase in knowledge (+12.1% vs. +11.6%; AOR = 1.1, 95% CI [0.7, 1.5]) 2 months after the cancer label in the intervention versus comparison site. Similar results were found 6 months after the cancer label for all three outcomes.

**Conclusions:**

In a real-world setting, cancer warning labels get noticed and increase knowledge that alcohol can cause cancer. Additional cancer label intervention studies are required that are not compromised by industry interference.

Cancer is a leading cause of disability and premature death globally (Global Burden of Disease Cancer Collaboration, 2017; Global Burden of Disease 2017 Causes of Death Collaborators, 2018). Estimates suggest that almost 40% of cancer cases are attributable to preventable risk factors, including alcohol (Poirier et al., 2019). Global alcohol consumption has increased 70% since 1990 (Manthey et al., 2019), and 2 billion people currently consume alcohol regularly. In 2012, alcohol caused approximately 480,000 cancer deaths, constituting 5.8% of total cancer deaths worldwide (Praud et al., 2016). Data show cancers are the predominant source of total alcohol-attributable deaths in higher income countries, particularly among those over age 50 (Global Burden of Disease 2016 Alcohol Collaborators, 2018). The ethanol in alcoholic beverages has been classified as a Group 1 carcinogen (the highest category of risk) since 1988 and is confirmed to be causally related to malignant tumors in at least seven sites, including high prevalence and often fatal cancers such as those of the colon and breast (International Agency for Research on Cancer[IARC], 2010a, 2010b). The causal relationship is accepted by expert groups, including the World Cancer Research Fund and the American Society for Clinical Oncology (LoConte et al., 2018; World Cancer Research Fund/American Institute for Cancer Research [AICR], 2007). Recent evidence extends the relationship between alcohol and increased cancer risk beyond heavy consumption to moderate and light drinking and to all types of alcohol including wine, beer, and spirits, concluding that there is no safe level of alcohol consumption (Choi et al., 2018; Global Burden of Disease 2016 Alcohol Collaborators, 2018). Drinking one bottle of wine per week is associated with an increase in absolute lifetime cancer risk equivalent to smoking 10 cigarettes a week for women and 5 for men (Hydes et al., 2019).

Supporting informed and safer alcohol use is now a crucial part of a public health strategy to reduce the risk of alcohol-related harms. Common policies to reduce population-level alcohol consumption and minimize harms involve restricting legal and physical access to alcohol. Fewer efforts have been made to inform consumers of alcohol-related health risks, particularly cancer, and this lack of awareness constitutes a significant public health need. A review of studies across 16 countries, for example, found only 13% in some jurisdictions are aware of the link between alcohol and cancer (Scheideler & Klein, 2018). In addition to drinkers not being informed, there are biases in how drinkers perceive alcohol-related risks. Public perceptions of alcohol are that it is less harmful than other drugs, and alcohol is largely not understood to be a carcinogen or is seen to be a risk only at high consumption levels (Buykx et al., 2015; Cheeta et al., 2018; *The Lancet,* 2018). The extent to which drinkers appreciate the magnitude of alcohol as a cancer risk, the more they may feel at risk, attend to low-risk drinking guidelines, and reconsider their drinking behaviors (Rosenberg et al., 2018). Research also shows public support for tightening alcohol control policies is stronger when the alcohol–cancer link is understood (Bates et al., 2018; Buykx et al., 2015; Martin et al., 2018; Weerasinghe et al., 2020).

Health warning labels are supported by the World Health Organization (WHO) for raising consumer awareness about the negative consequences of alcohol (WHO, 2010, 2017). In contrast to other information-based interventions, alcohol labels are unique in that drinkers are exposed to health messages at key points of contact—the point-of-purchase and -pour. Extensive international research examining warning labels on tobacco packages indicates that well-designed warning labels, particularly labels on the front of packages, which are large in size with specific health messages that rotate and with color pictures, influence behavior by gaining consumers’ attention, eliciting aversive reactions, and keeping the message in consumers’ minds (Brewer et al., 2019; Hammond, 2011; Hiilamo et al., 2014; Noar et al., 2017). Labels are appealing because of their low cost to regulators, unparalleled reach among drinkers, and higher exposure among the heaviest drinkers (Greenfield, 1997). Lab and online studies testing alcohol label messages show that cancer warnings are most effective for educating drinkers about the seriousness of alcohol-related health harms and strengthening intentions to reduce alcohol intake compared with other health messages (Al-Hamdani & Smith, 2015, 2017; Jongenelis, 2018; *The Lancet,* 2018). More than 47 countries now mandate alcohol labels. Most mandate labels with vague statements of risk, or cautioning about the risk of drinking alcohol during pregnancy or when operating a motor vehicle (WHO, 2018). Only two countries currently require labels with a cancer warning. Since 2017, alcohol manufacturers in South Korea are required to choose one of three messages, two of which cite cancer risk. Ireland passed legislation in late 2018 mandating cancer warnings on alcohol product labels. With limited uptake worldwide, the effectiveness of cancer warning labels on alcohol remains largely unstudied (Martin-Moreno et al., 2013).

This study is the first real-world study to test if cancer warning labels on alcohol containers are an effective tool for increasing population awareness that alcohol can cause cancer. More specifically, this study tested the initial and continued effects of cancer warning labels on drinkers’ recall of the cancer warning and knowledge that alcohol can cause cancer. In addition, this study describes support for health warning labels on alcohol containers and assesses the association between knowledge and support for labels.

## Method

### Alcohol label intervention

**Figure 1.**
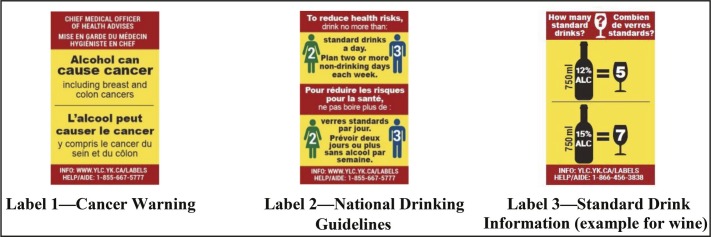
Intervention alcohol warning labels (actual size 5.0 cm × 3.2 cm): Alcohol containers sold in the liquor store in the intervention site displayed only one of the labels at any one time.

As shown in Figure 1, the alcohol label intervention consisted of three labels stating (a) a cancer warning with specific references to breast and colon cancers, (b) national drinking guidelines, and (c) standard drink information (four separate labels were developed for wine, spirits, coolers, and beer). Label content, size, and format were informed by evidence as well as consultations with local and international health experts and community stakeholders (Blackwell et al., 2018; Hammond, 2011; Hobin et al., 2018; Martin-Moreno et al., 2013; Noar et al., 2017; Pettigrew et al., 2016; Strahan et al., 2002; Vallance et al., 2018; Wettlaufer, 2018; WHO, 2017). The labels were relatively large to make them easily noticed and read, were full color with a bright yellow background and red border so they stood out on the container, and had messages providing new information. They were rotated to avoid wear out. Label messages were provided in Canada’s two official languages, English and French. Consistent with evidence for effective labeling (Hammond, 2011; Martin-Moreno et al., 2013), a parallel social marketing and awareness campaign was designed that included in-store signage, handouts, a website, toll-free helpline, and radio spots to augment the label messages.

### Study design

A pre–post quasi-experimental study with comparison group was designed. The intervention site (Whitehorse, Yukon, Canada) was recruited to apply intervention alcohol labels on all alcohol containers, except select local and single-serve beer and cider, in its one government-owned liquor store for an 8-month period. The comparison site (Yellowknife, Northwest Territories, Canada) consisted of two government-owned liquor stores that continued usual labeling practices. These stores are the only government monopoly liquor stores and account for almost all legal off-premise alcohol sales in both cities (Government of Northwest Territories, 2017; Government of Yukon, 2017). Yukon and Northwest Territories were recruited to participate in this experiment because they are currently the only jurisdictions in Canada to require any kind of alcohol warning label. Since 1991, they have used after-market labels on alcohol containers to caution consumers about the risk of drinking while pregnant, with an additional warning in Northwest Territories about drinking and operating machinery and general health concerns (Government of Northwest Territories, 2017; Government of Yukon, 2017; Greenfield, 1997).

### Timing of data collections

Two waves of surveys were scheduled in the intervention and comparison sites before and after the intervention labels were implemented in the intervention site. Wave 1 surveys were conducted in both sites over a 6-week period starting May 2017, approximately 4 months before the labels were implemented in the intervention site. Wave 2 surveys were scheduled over a 6-week period starting May 2018, 8 months after implementation. Starting November 20, 2017, two of the intervention labels, the cancer warning and national drinking guidelines, were applied to alcohol containers in the liquor store in the intervention site. The standard drink labels were to be introduced shortly thereafter. However, only 1 month into the 8-month alcohol label intervention period, the government for the intervention site halted their participation in the study owing to significant pressure from representatives of Canada’s national alcohol producers and stopped applying labels (Austen, 2018; Vallance et al., 2020).

**Figure 2.**
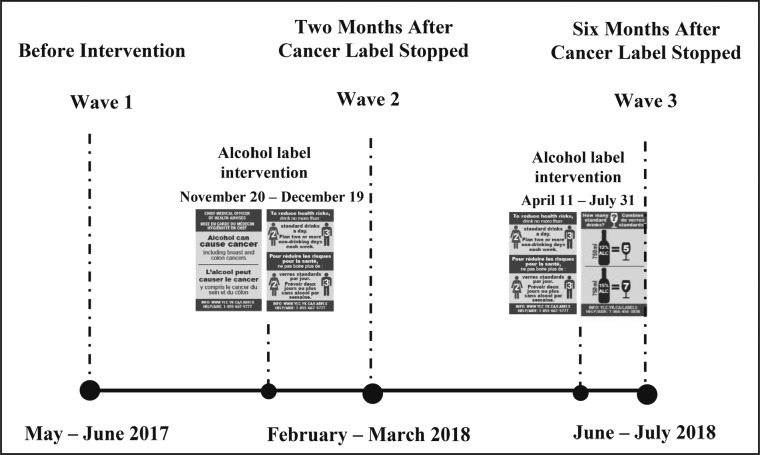
Modified study design after alcohol industry interference

Based on remaining label stock, approximately 47,000 cancer warning labels and 53,000 national drinking guidelines labels were applied to alcohol containers within the 1-month period. As a result of the unexpected interruption, the study design was modified (Figure 2). Wave 2 surveys were conducted starting February 2018, 2 months after the government paused their participation, in order to capture any impact of the shortened intervention. In April 2018, the government resumed their participation in the label intervention, under the condition that the cancer warning label be excluded from rotation. Thus, the labels containing the national drinking guidelines were reinstated in the liquor store in the intervention site starting April 12, 2018, and the standard drink labels followed starting May 28, 2018, up to the end of July 31, 2018. A third wave of surveys (Wave 3) was conducted starting June 2018 to the end of the intervention period in July 2018 to assess the impact of the two labels with drinking guidelines and standard drink information as well as the potential continued effect of the omitted cancer warning label. The project website and toll-free number were implemented in November 2017, at the time of the original intervention launch; however, in-store posters, point-of-sale materials, and radio spots were not implemented owing to industry interference. Full details of the alcohol labeling intervention and study design are described in Vallance et al. (2020).

### Recruitment and survey procedures

In Wave 1, a prospective cohort of adult drinkers was recruited by trained research assistants as customers exited the liquor stores in the intervention and comparison sites. A standard intercept technique was used of approaching every person who passed a pre-identified landmark in the liquor store. Eligibility for the survey was established through a screening tool. Eligible participants were given study information and a consent form. Consenting participants were instructed to complete the survey on a tablet independently, without assistance. Participants were offered a gift card as remuneration for their time. In Waves 2 and 3, participants who provided their contact information were emailed survey instructions, a unique survey link, and an INTERAC e-transfer as remuneration. In addition, because of attrition in Waves 2 and 3, the sample was replenished using Wave 1 recruitment and survey procedures in the liquor stores in both the intervention and comparison sites. All survey periods continued for 6 weeks, the survey was approximately 18 minutes in length, and survey measures were consistent across waves and sites. All procedures were approved by the Research Ethics Boards at Public Health Ontario (ID 2017-010.04) and the University of Victoria (Protocol 17-161).

### Participants

Participants were adults of legal drinking age (≥19), residents of either the intervention or comparison cities, and at the time of recruitment were current drinkers (consumed one or more alcoholic drinks in the past 30 days), purchased alcohol at the liquor store, and did not self-report being pregnant or breastfeeding.

### Measures

#### Noticing labels.

To assess noticing alcohol labels, participants were asked if they had seen any warning labels on bottles or cans of beer, wine, distilled spirits, coolers, or ciders. Responses were dichotomized as *noticed* and *did not notice/don’t know.* The measure at Wave 1 was anchored with 6 months prior, the measure at Wave 2 from November before follow-up, and Wave 3 from April before follow-up.

#### Unprompted and prompted recall.

Among those who indicated noticing warning labels, participants were first asked an open-ended question to assess what messages they saw on the warning labels on alcohol containers without being prompted. Subsequently, participants were asked if they saw any of the following messages on alcohol containers and asked to check all that apply. Response options included *alcohol and cancer, low-risk drinking guidelines, number of standard drinks in bottles or cans, alcohol may be an addictive drug, alcohol and liver disease, alcohol and trauma, alcohol and fetal alcohol spectrum disorder,* and *drinking alcohol and driving a car or operating machinery.* Both recall measures were anchored similarly to the “noticing labels” measure above. For the unprompted recall measure, a research assistant blinded to experimental conditions coded each response. Any mention of “cancer” was coded as recall of the cancer label. Ambiguous responses were reviewed by and discussed with a second coder to reach consensus.

#### Knowledge of alcohol as a carcinogen.

Knowledge that alcohol can cause cancer was assessed by asking participants, “Based on what you know or believe, can drinking alcohol cause . . . ?” and this item was asked for breast cancer, liver disease, the flu, and [when pregnant] harm to unborn babies. Response options included “yes,” “no,” or “don’t know,” and responses were dichotomized as “yes” versus “no/don’t know.” Only responses to the cancer item are reported here.

### Support for health warning labels on alcohol containers

To assess support for health warning labels on alcohol containers, participants were asked the extent to which they agree or disagree with the statement, “Cans and bottles of alcoholic beverages should be labeled with warnings describing the link between alcohol and diseases, such as cancer.” Responses were measured on a 5-point Likert scale (1 = *strongly disagree,* 5 = *strongly agree*) and included “don’t know” and “prefer not to say” as options.

### Sociodemographic characteristics

Sociodemographic measures included age, sex, ethnicity (White, Aboriginal, and other/don’t know/prefer not to say/missing), education (low [completed high school or less], medium [completed trades or college certificate, some university or university certificate below a bachelor’s degree], high [university degree or post-graduation], and unknown [don’t know/prefer not to say/missing]), and income (low [<CAD$30,000], medium [CAD$30,000–$59,999], high [CAD≥$60,000], and unknown [don’t know/prefer not to say/missing]).

### Other covariates

Exposure to sources of information on alcohol-related health risks was measured by asking respondents if they had noticed advertising or information that talks about the dangers of drinking alcohol, or encourages people to cut down or stop drinking, in six specific locations (*yes* vs. *no/don’t know/prefer not to say*). Health literacy was assessed using the Newest Vital Sign assessment tool (Weiss et al., 2005) and responses were categorized as *limited* (≤1 correct responses), *possibility of limited* (2–3 correct responses), *adequate literacy* (4–6 correct responses), and *unknown* (don’t know/prefer not to say/missing). Alcohol use was measured using the quantity/frequency method (Heeb & Gmel, 2005). Participants were asked to indicate how often they drank alcoholic beverages in the past 6 months and how many drinks they usually drank per occasion. Responses were combined to provide a mean number of drinks per week and categorized using Canada’s national drinking guidelines as follows: *low* (≤10 for females/15 for males per week), *risky* (11–19/16–29 per week), *high* (≥20/30 per week) (Butt et al., 2011), and *unknown* (don’t know/prefer not to say/missing). Last, a time-in-sample variable was created to adjust for participants who participated in one, two, or all three survey waves.

### Statistical analysis

Logistic regression models using generalized estimating equations (GEE) were applied to examine the impact of labels on the three main outcomes. GEE models can account for a mix of within-subject correlation that arises from the cohort participants being asked the same questions over multiple survey waves plus the replenishment sample. Difference-in-difference (DID) terms were added to each model to assess the change in outcomes across waves and between sites. The DID terms included an interaction between wave and site, which allowed for a formal test of whether the pattern of change over time in the intervention site was significantly different from the comparison site. Sociodemographic variables and the remaining covariates were included in all models. Education, income, and health literacy were found to be correlated; thus, to improve the stability of the models, only education was used. The exposure to information measures were combined into one variable that indicates if participants were exposed to any source of alcohol information in the media; however, the variable did not make a difference in the results because of the lack of variability across sites, and the final models did not adjust for exposure to media to avoid over-adjusting. “Prefer not to say” and “missing” responses were removed from all outcome measures. As per agreement with the local territorial government partners, ethnicity, defined as White versus other (Aboriginal/other/don’t know/prefer not to say/missing), is included in the sample description and adjusted for in the analyses, but not reported in the results. Support for health warning labels on alcohol containers is reported descriptively, and the overall association between knowledge that alcohol is a carcinogen and support for health warning labels across sites and survey waves is assessed using a GEE model. As previous literature has identified qualitative differences between individuals who respond “yes,” “no,” and “don’t know” to items assessing knowledge that alcohol can cause cancer (Wiseman & Klein, 2019), a sensitivity analysis tested the effect of modeling these responses separately using a GEE model with a multinomial distribution. Last, three-way interactions were tested for each of the three main outcomes across site, wave, and health literacy and alcohol drinking levels. Health literacy was dichotomized as adequate literacy versus all other options, and drinking level was dichotomized as low versus all other options. All analyses were conducted using SAS Version 9.3 (SAS Institute Inc., Cary, NC).

## Results

In total, 2,049 unique participants completed at least one of the three surveys. According to AAPOR #4, response rates were 8.9% in the intervention and 8.0% in the comparison sites (American Association for Public Opinion Research, 2011). Overall, 53.2% participants were retained at Wave 2, and 47.5% at Wave 3. Table 1 presents the sample characteristics by site at time of recruitment, and Table 2 indicates the sources of information on the dangers of alcohol in the media by wave and site.

**Table 1.**
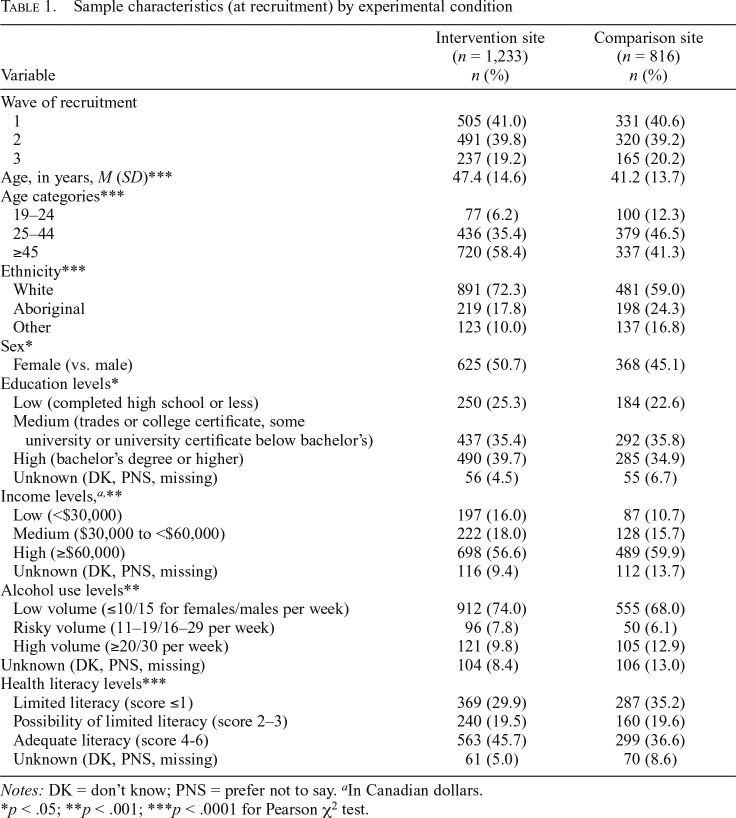
Sample characteristics (at recruitment) by experimental condition

Variable	Intervention site (*n* = 1,233) *n* (%)	Comparison site (*n* = 816) *n* (%)
Wave of recruitment		
1	505 (41.0)	331 (40.6)
2	491 (39.8)	320 (39.2)
3	237 (19.2)	165 (20.2)
Age, in years, *M* (*SD*)***	47.4 (14.6)	41.2 (13.7)
Age categories***		
19–24	77 (6.2)	100 (12.3)
25–44	436 (35.4)	379 (46.5)
≥45	720 (58.4)	337 (41.3)
Ethnicity***		
White	891 (72.3)	481 (59.0)
Aboriginal	219 (17.8)	198 (24.3)
Other	123 (10.0)	137 (16.8)
Sex*		
Female (vs. male)	625 (50.7)	368 (45.1)
Education levels*		
Low (completed high school or less)	250 (25.3)	184 (22.6)
Medium (trades or college certificate, some university or university certificate below bachelor’s)	437 (35.4)	292 (35.8)
High (bachelor’s degree or higher)	490 (39.7)	285 (34.9)
Unknown (DK, PNS, missing)	56 (4.5)	55 (6.7)
Income levels,^a,^**		
Low (<$30,000)	197 (16.0)	87 (10.7)
Medium ($30,000 to <$60,000)	222 (18.0)	128 (15.7)
High (≥$60,000)	698 (56.6)	489 (59.9)
Unknown (DK, PNS, missing)	116 (9.4)	112 (13.7)
Alcohol use levels**		
Low volume (≤10/15 for females/males per week)	912 (74.0)	555 (68.0)
Risky volume (11–19/16–29 per week)	96 (7.8)	50 (6.1)
High volume (≥20/30 per week)	121 (9.8)	105 (12.9)
Unknown (DK, PNS, missing)	104 (8.4)	106 (13.0)
Health literacy levels***		
Limited literacy (score ≤1)	369 (29.9)	287 (35.2)
Possibility of limited literacy (score 2–3)	240 (19.5)	160 (19.6)
Adequate literacy (score 4-6)	563 (45.7)	299 (36.6)
Unknown (DK, PNS, missing)	61 (5.0)	70 (8.6)

*Notes:* DK = don’t know; PNS = prefer not to say.

^a^ In Canadian dollars.

* *p* < .05;

** *p* < .001;

*** *p* < .0001 for Pearson χ^2^ test.

**Table 2.**
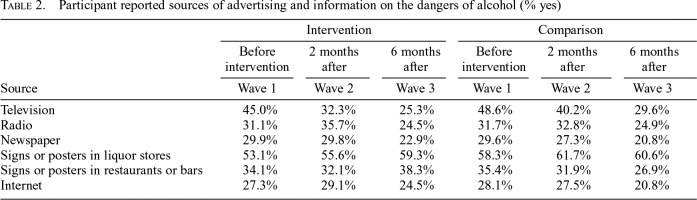
Participant reported sources of advertising and information on the dangers of alcohol (% yes)

Source	Intervention			Comparison		
	Before intervention	2 months after	6 months after	Before intervention	2 months after	6 months after
	Wave 1	Wave 2	Wave 3	Wave 1	Wave 2	Wave 3
Television	45.0%	32.3%	25.3%	48.6%	40.2%	29.6%
Radio	31.1%	35.7%	24.5%	31.7%	32.8%	24.9%
Newspaper	29.9%	29.8%	22.9%	29.6%	27.3%	20.8%
Signs or posters in liquor stores	53.1%	55.6%	59.3%	58.3%	61.7%	60.6%
Signs or posters in restaurants or bars	34.1%	32.1%	38.3%	35.4%	31.9%	26.9%
Internet	27.3%	29.1%	24.5%	28.1%	27.5%	20.8%

The proportion of respondents who noticed the labels was high across all three survey waves in both the intervention (Wave 1 = 80.4%, Wave 2 = 76.7%, Wave 3 = 80.5%) and comparison (Wave 1 = 87.0%, Wave 2 = 78.5%, Wave 3 = 72.9%) sites.

**Figure 3.**
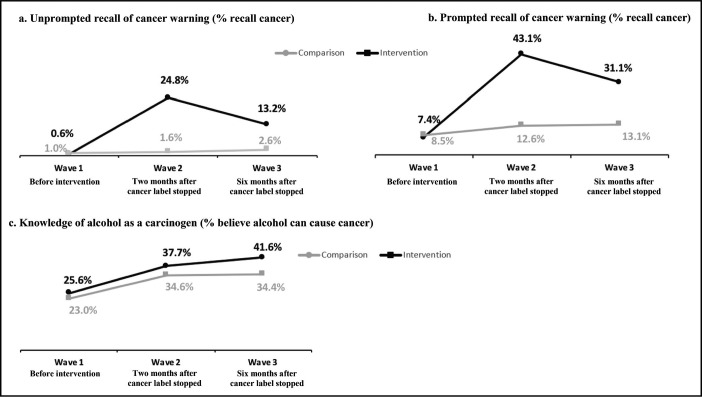
(a–c). Percentage of participants recalling cancer warning, unprompted and prompted, and knowledge of alcohol as a carcinogen across survey waves in intervention and comparison sites

Unprompted recall of the cancer warning message increased to a greater extent between Wave 1 (before the cancer warning label) and Wave 2 (2 months after the cancer warning label was stopped) in the intervention versus comparison site (+24.2% vs. 0.6%; adjusted odds ratio [AOR] = 32.7, 95% CI [5.4, 197.7]), and between Wave 1 and Wave 3 (6 months after the cancer warning label was stopped) (+12.6% vs. +1.6%; AOR = 8.8, 95% CI [1.6, 49.4]) (Table 3; Figure 3a). Results of prompted recall also increased to a greater extent between Waves 1 and 2 in the intervention versus the comparison site (+35.7% vs. 4.1%; AOR = 6.2, 95% CI [3.6, 10.9]), and between Waves 1 and 3 (+23.7% vs. +4.6%; AOR = 3.5, 95% CI [2.0, 6.2]) (Table 3; Figure 3b).

**Table 3.**
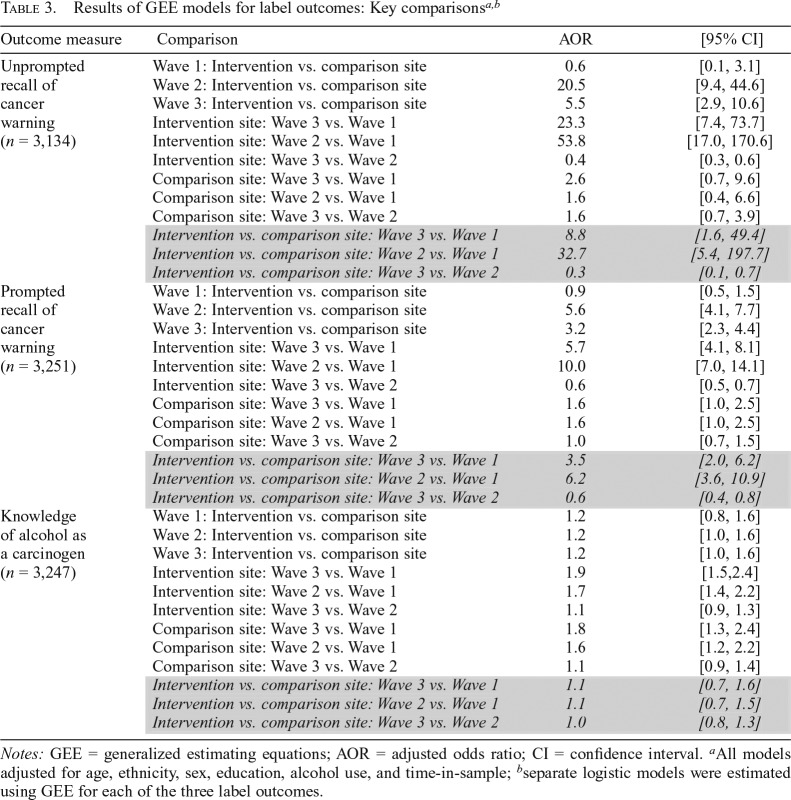
Results of GEE models for label outcomes: Key comparisons^*a**,**b*^

Outcome measure	Comparison	AOR	[95% CI]
Unprompted recall of cancer warning (*n* = 3,134)	Wave 1: Intervention vs. comparison site	0.6	[0.1, 3.1]
	Wave 2: Intervention vs. comparison site	20.5	[9.4, 44.6]
	Wave 3: Intervention vs. comparison site	5.5	[2.9, 10.6]
	Intervention site: Wave 3 vs. Wave 1	23.3	[7.4, 73.7]
	Intervention site: Wave 2 vs. Wave 1	53.8	[17.0, 170.6]
	Intervention site: Wave 3 vs. Wave 2	0.4	[0.3, 0.6]
	Comparison site: Wave 3 vs. Wave 1	2.6	[0.7, 9.6]
	Comparison site: Wave 2 vs. Wave 1	1.6	[0.4, 6.6]
	Comparison site: Wave 3 vs. Wave 2	1.6	[0.7, 3.9]
	*Intervention vs. comparison site: Wave 3 vs. Wave 1*	*8.8*	*[1.6, 49.4]*
	*Intervention vs. comparison site: Wave 2 vs. Wave 1*	*32.7*	*[5.4, 197.7]*
	*Intervention vs. comparison site: Wave 3 vs. Wave 2*	*0.3*	*[0.1, 0.7]*
Prompted recall of cancer warning (*n* = 3,251)	Wave 1: Intervention vs. comparison site	0.9	[0.5, 1.5]
	Wave 2: Intervention vs. comparison site	5.6	[4.1, 7.7]
	Wave 3: Intervention vs. comparison site	3.2	[2.3, 4.4]
	Intervention site: Wave 3 vs. Wave 1	5.7	[4.1, 8.1]
	Intervention site: Wave 2 vs. Wave 1	10.0	[7.0, 14.1]
	Intervention site: Wave 3 vs. Wave 2	0.6	[0.5, 0.7]
	Comparison site: Wave 3 vs. Wave 1	1.6	[1.0, 2.5]
	Comparison site: Wave 2 vs. Wave 1	1.6	[1.0, 2.5]
	Comparison site: Wave 3 vs. Wave 2	1.0	[0.7, 1.5]
	*Intervention vs. comparison site: Wave 3 vs. Wave 1*	*3.5*	*[2.0, 6.2]*
	*Intervention vs. comparison site: Wave 2 vs. Wave 1*	*6.2*	*[3.6, 10.9]*
	*Intervention vs. comparison site: Wave 3 vs. Wave 2*	*0.6*	*[0.4, 0.8]*
Knowledge of alcohol as a carcinogen (*n* = 3,247)	Wave 1: Intervention vs. comparison site	1.2	[0.8, 1.6]
	Wave 2: Intervention vs. comparison site	1.2	[1.0, 1.6]
	Wave 3: Intervention vs. comparison site	1.2	[1.0, 1.6]
	Intervention site: Wave 3 vs. Wave 1	1.9	[1.5,2.4]
	Intervention site: Wave 2 vs. Wave 1	1.7	[1.4, 2.2]
	Intervention site: Wave 3 vs. Wave 2	1.1	[0.9, 1.3]
	Comparison site: Wave 3 vs. Wave 1	1.8	[1.3, 2.4]
	Comparison site: Wave 2 vs. Wave 1	1.6	[1.2, 2.2]
	Comparison site: Wave 3 vs. Wave 2	1.1	[0.9, 1.4]
	*Intervention vs. comparison site: Wave 3 vs. Wave 1*	*1.1*	*[0.7, 1.6]*
	*Intervention vs. comparison site: Wave 2 vs. Wave 1*	*1.1*	*[0.7, 1.5]*
	*Intervention vs. comparison site: Wave 3 vs. Wave 2*	*1.0*	*[0.8, 1.3]*

*Notes:* GEE = generalized estimating equations; AOR = adjusted odds ratio; CI = confidence interval.

^a^ All models adjusted for age, ethnicity, sex, education, alcohol use, and time-in-sample;

^b^ separate logistic models were estimated using GEE for each of the three label outcomes.

Knowledge that alcohol can cause cancer was low in Wave 1 in both the intervention (25.6%) and comparison (23.0%) sites, and increased in Waves 2 and 3 in both sites (Figure 3c). Although knowledge of alcohol as a carcinogen increased in both sites, the DID analyses revealed a 10% greater increase in knowledge in the intervention relative to the comparison site between both Waves 1 and 2 (+12.1% vs. +11.6%; AOR = 1.1, 95% CI [0.7, 1.5]) and Waves 1 and 3 (+16.0% vs. 11.4%; AOR = 1.1, 95% CI [0.7, 1.6]; Table 3). Results of the models indicate differences ranging from a 30% decrease, a small negative change, to a 50%–60% increase, a substantial positive change. In the sensitivity analyses, results of DID comparisons evaluating the labels’ effect on knowledge of alcohol as a carcinogen between intervention and comparison sites for the responses “yes,” “no,” and “don’t know” separately, indicate similar trends when comparing “yes” versus “no” responses and “yes” versus “don’t know” responses (Supplemental Table A). (Supplemental material is available as an online-only addendum to this article on the journal’s website.)

To further confirm the contribution of the cancer warning labels to consumer knowledge, a GEE model with a binomial distribution estimating the relationship between recall, either unprompted or prompted, and knowledge of alcohol as a carcinogen across the three waves was conducted, adjusting for sociodemographics and other covariates, including exposure to sources of information in the media. The results indicated that those who recalled the cancer message had 2.3 greater odds of knowing alcohol can cause cancer (AOR = 2.3, 95% CI [1.9, 2.7]).

Results of the three-way interactions across site, wave, and each of health literacy and drinking level were not statistically significant for prompted and unprompted recall and knowledge that alcohol can cause cancer (Supplemental Table B).

**Figure 4.**
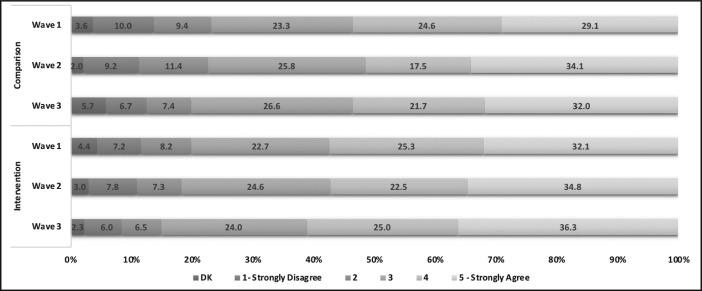
Degree of support for health warning labels on alcohol containers across survey waves in intervention and comparison sites (% of participants; n = 2,022 unique participants). DK = don’t know.

Last, the degree to which participants support health warning labels on alcohol containers is presented in Figure 4, ordered from *strongly disagree* to *strongly agree.* Most participants reported agreeing to strongly agreeing with health warning labels on alcohol containers in the intervention (Wave 1 = 57.4%; Wave 2 = 57.3%; Wave 3 = 61.3%) and comparison (Wave 1 = 53.7%; Wave 2 = 51.6%; Wave 3 = 53.7%) sites. The results also indicated that those who know alcohol can cause cancer are 1.6 times more likely to support health warning labels relative to those who do not know, adjusting for sociodemographics and other covariates (AOR = 1.6, 95% CI [1.38, 1.89]).

## Discussion

It is argued by the alcohol industry that drinkers are adequately informed about the health risks of alcohol and that warning labels do not work (Éduc’alcool, 2019; Kane, 2018). Yet, international health experts recommend health warning labels on alcohol as an increasingly popular public health strategy for providing information to consumers about the various health risks of alcohol use (Greenfield, 1997; WHO, 2010, 2017, 2018). This is the first study to experimentally examine the population-level effects of a cancer warning label on alcohol containers in a real-world setting. Label effectiveness is influenced by the extent to which consumers notice, recall, and understand the label information and eventually make the decision to consume the product in a given situation (IARC, 2008). Despite the interrupted and briefer-than-intended application of the cancer warning labels in this study, consumers noticed the labels. Two months after the cancer warning labels, almost 25% of participants exposed to the intervention recalled the cancer warning message unprompted, and recall rose to 43% when prompted. As expected, recall decreased 6 months after the cancer warning labels were removed, demonstrating intervention specificity.

Greater awareness of the cancer risks associated with alcohol is thought to be a potentially effective strategy for strengthening public acceptance of alcohol control measures and awareness of national drinking guidelines (Bates et al., 2018; Buykx et al., 2015; Rosenberg et al., 2018; Weerasinghe et al., 2020). Indeed, the results in the current article indicate a positive association between knowledge and support for alcohol labels. In addition, a separate analysis conducted as part of the larger study and reported in Weerasinghe et al. (2020) suggests that increases in individual-level knowledge that alcohol can cause cancer are associated with almost two times greater likelihood of supporting minimum unit alcohol pricing policies. In the current article, before the label intervention, knowledge of the alcohol–cancer link was approximately 25% in both sites, low yet consistent with previous estimates in Canada (Public Health Ontario, 2017). Knowledge grew to 41.6% in the intervention site, increasing 12.1% 2 months after, and a further 3.9% 6 months after the warning label was no longer being applied to containers, demonstrating the immediate and continued effects of the cancer labels. This continued effect may be the result of left-over cancer labels on containers still available for purchase in the liquor store or already purchased and served at home or in restaurants. Overall, the 10% greater increase in knowledge in the intervention relative to the comparison site is a modest yet meaningful population-level effect. Results also revealed that drinkers who recalled the cancer label message were 2.3 times more likely to know that alcohol can cause cancer, after controlling for sociodemographic variables, alcohol consumption level, and exposure to sources of information in the media. These findings provide evidence that crucial early processes are required for labels to be an effective means of communicating health information to drinkers.

In this study, differences in knowledge of the alcohol–cancer link between the intervention and comparison sites were attenuated because of the sudden increase in knowledge in the comparison site during the intervention period. The surge in knowledge in the comparison site can likely be explained by the substantial national and international media coverage of the alcohol industry’s efforts to stop the alcohol label intervention, specifically the cancer warning label (Austen, 2018; Vallance et al., 2020). The media coverage also may have augmented interest in the label intervention in the intervention site; however, the social marketing and awareness campaign that was originally intended to supplement the alcohol labels in the intervention site, but was not implemented owing to the interruption by the alcohol industry, would have served a similar purpose. This study did include survey measures to control for participant exposure to other sources of alcohol information that may have confounded the effect of the labels during the intervention period; however, there were no measurable differences in these variables between sites, as shown in Table 2, and controlling for these variables in the analyses did not alter the main results. It is plausible that the information measures did not detect differences in media exposure in this study because the measures did not specifically assess media coverage of alcohol labels or the alcohol industry, but instead assessed exposure to advertising or information that talks about the dangers of drinking alcohol, or encourages people to cut down or stop drinking. Additional cancer label intervention studies are required that are not compromised by industry interference.

The impacts of the cancer warning labels on awareness and knowledge observed in this study are comparable to the population-level effects of two mass media alcohol and cancer campaigns in Australia and the United Kingdom, both of which were multicomponent and likely relatively expensive (Dixon et al., 2015; Martin et al., 2018). The evaluations of these two campaigns lack comparison groups, which limits measuring the contribution of secular trends in the absence of the campaigns; nevertheless, these examples demonstrate the unique benefit of labels and underscore the potential cost-effectiveness of warning labels on alcohol containers that could be introduced at little or no cost to governments.

Results of the current real-world experimental study indicate that cancer warning labels can be an effective intervention for communicating information across subpopulations, as we found no evidence that the label intervention differentially affected participants with varying health literacy and drinking levels. However, the results from this single study should be interpreted cautiously because existing lab-based experimental studies, using diverse study designs and methods to test cancer warnings, (a) found associations between outcomes (e.g., knowledge of alcohol-related health risks, self-reported drinking behavior) and participant characteristics (e.g., sex, drinking level; Miller et al., 2016; Pettigrew et al., 2014, 2016), (b) did not examine the differential impacts of cancer warnings by participant characteristics (Al-Hamdani et al., 2015, 2017; Blackwell et al.,2018; Stafford & Salmon, 2017; Wigg et al., 2016), or (c) did not find evidence to suggest differential impacts for a cancer warning by participant characteristics (Jongenelis et al., 2018). Although the U.S. alcohol warning label does not include a cancer warning message, a relatively large real-world evaluation of this label found that label awareness and recall were highest among heavy drinkers, pregnant women, and young adults (Greenfield et al., 1999; Kaskutas & Greenfield, 1992). More research is required to better understand the impact of alcohol warning labels by receiver characteristics as the effectiveness and equity of information-based interventions have been questioned and, in some countries, the concentrations of alcohol-related hospitalizations and mortality are higher among groups of lower socioeconomic status despite reporting intake levels similar to or comparatively less than more affluent groups (Katikireddi et al., 2017; Probst et al., 2014).

Our results also show that health warning labels on alcohol are unlikely to be received negatively among drinkers, with most participants in both sites supporting health warning labels linking alcohol and diseases, specifically cancer, on alcohol containers. Similar outcomes were observed in previous studies, which reported that responses to cancer-related alcohol warning labels were generally positive (Miller et al., 2016; Pettigrew et al., 2014). Further research is needed to determine if repeated exposure to cancer warning labels on alcohol containers over a longer uninterrupted period may strengthen their impact.

### Limitations

This study has several limitations. First, the cancer warning label was halted 1 month into the 8-month intervention period with a 2-month lag between labeling and the Wave 2 survey and a 6-month lag for the Wave 3 survey. This briefer-than-intended intervention period and gap in follow-up survey waves may have attenuated the cancer warning labels’ influence, and uncertainty remains about their longer term impact. Next, the DID analyses estimating the alcohol label intervention’s effect on changes in knowledge between sites over time did not reach levels of conventional statistical significance. This is likely because of the small sample sizes in both sites, which in turn produced wide confidence intervals and less precise estimates. It is reasonable to assume that these first two limitations (shortened intervention period and small sample sizes), in addition to the media contamination in the comparison site discussed above, led to smaller differences in knowledge between the intervention and comparison sites over time and biased the DID results toward the null. Thus, as recommended, the point estimates and upper and lower limits are described in the results, and a range of potential explanations is discussed (Amrhein et al., 2019).

Next, the study cannot provide representative estimates of the population, because participants were recruited from liquor stores in city centers using systematic recruitment methods. However, given that the stores from which the customers were recruited are virtual monopolies for the off-premise sale of alcohol in both experimental sites, they will have been broadly representative of persons purchasing alcohol in those cities. One bias would have been toward heavier drinkers more likely to buy alcohol frequently, an important target group for warning label interventions. Health knowledge can be assessed several ways. This study used only one measure specific to breast cancer to test participants’ knowledge of the alcohol–cancer link. Previous studies examining knowledge of alcohol-related cancers often report the lowest levels for breast cancer relative to other types, such as liver and colon (Buykx et al, 2015; Scheideler & Klein, 2018; *The Lancet,* 2018). Future research could use more comprehensive measures with a higher threshold of knowledge by asking respondents to recall alcohol-related diseases unprompted, or to estimate the likelihood of alcohol-related diseases. Last, given that the alcohol label intervention consists of three complementary label messages, it is difficult to attribute the changes in consumer knowledge of the link between alcohol and cancer solely to the cancer warning. It is possible the other two label messages prompted consumers to reconsider their alcohol drinking and potential harms.

### Conclusions

Despite the brief duration of the intervention, the study results support the use of cancer warning labels on alcohol containers as a strategy to increase knowledge of alcohol as a cancer risk, a stated goal of international alcohol control efforts (LoConte et al., 2018; WHO, 2010, 2017; World Cancer Research Fund/AICR, 2007). Overall, drinkers exposed to the label intervention recalled the cancer warning message, and the warning label increased knowledge of the alcohol–cancer link. Increases in knowledge that alcohol can cause cancer in the comparison site and, to some degree, in the intervention site likely reflect the considerable public interest in the media coverage of the alcohol industry’s actions to disrupt the study and remove the cancer warning label. The alcohol industry’s opposition to cancer warnings on containers, coupled with the broad public support for health warnings on alcohol, highlights the importance of mandatory alcohol labeling to ensure that consumers are adequately informed.

## Acknowledgments

The authors acknowledge all of our research assistants who helped with data collections, as well as the liquor control boards, health and social services, and community partners in Yukon and Northwest Territories for their commitment and support in developing and executing this research. Special thanks also go to Mark Petticrew and Melanie Wakefield for their expertise and guidance.

## References

[B1] Al-Hamdani M. Smith S. 2015 Alcohol warning label perceptions: Emerging evidence for alcohol policy *Canadian Journal of Public Health* 106 e395 e400 doi:10.17269/CJPH.106.5116 26680431 10.17269/CJPH.106.5116 PMC6972042

[B2] Al-Hamdani M. Smith S. M. 2017 Alcohol warning label perceptions: Do warning sizes and plain packaging matter? *Journal of Studies on Alcohol and Drugs* 78 79 87 doi:10.15288/jsad.2017.78.79 27936367 10.15288/jsad.2017.78.79

[B3] American Association for Public Opinion Research 2011 *Standard definitions: Final dispositions of case codes and outcome rates for surveys (7th ed.)* Retrieved from https://www.esomar.org/uploads/public/knowledge-and-standards/codes-and-guidelines/ESOMAR_Standard-Definitions-Final-Dispositions-of-Case-Codes-and-Outcome-Rates-for-Surveys.pdf

[B4] Amrhein V. Greenland S. McShane B. 2019 Scientists rise up against statistical significance *Nature* 567 305 307 doi:10.1038/d41586-019-00857-9 30894741 10.1038/d41586-019-00857-9

[B5] Austen I. 2018, January 6) Yukon government gives in to liquor industry on warning label experiment *The New York Times* Retrieved from https://www.nytimes.com/2018/01/06/world/canada/yukon-liquor-alcohol-warnings.html

[B6] Bates S. Holmes J. Gavens L. de Matos E. G. Li J. Ward B. Buykx P. 2018 Awareness of alcohol as a risk factor for cancer is associated with public support for alcohol policies *BMC Public Health* 18 Article no. 688. doi:10.1186/s12889-018-5581-8 10.1186/s12889-018-5581-8 PMC5987582 29866082

[B7] Blackwell A. K. M. Drax K. Attwood A. S. Munafò M. R. Maynard O. M. 2018 Informing drinkers: Can current UK alcohol labels be improved? *Drug and Alcohol Dependence* 192 163 170 doi:10.1016/j.drugalcdep.2018.07.032 30265999 10.1016/j.drugalcdep.2018.07.032 PMC6204577

[B8] Brewer N. T. Parada H. Hall M. G. Boynton M. H. Noar S. M. Ribisl K. M. 2019 Understanding why pictorial cigarette pack warnings increase quit attempts *Annals of Behavioral Medicine: A Publication of the Society of Behavioral Medicine* 53 232 243 doi:10.1093/abm/kay032 29850764 10.1093/abm/kay032 PMC6265120

[B9] Butt P. Beirness D. Stockwell T. Gliksman L. Paradis C. 2011 *Alcohol and health in Canada: A summary of evidence and guidelines for low-risk drinking* Ottawa, Ontario Canadian Centre on Substance Abuse Retrieved from http://www.ccsa.ca/Resource%20Library/2011-Summary-of-Evidence-and-Guidelines-for-Low-Risk%20Drinking-en.pdf

[B10] Buykx P. Li J. Gavens L. Lovatt M. Gomes de Matos E. Holmes J. Meier P. 2015 *An investigation of public knowledge of the link between alcohol and cancer* University of Sheffield and Cancer Research UK Retrieved from https://www.cancerresearchuk.org/sites/default/files/an_investigation_of_public_knowledge_of_the_link_between_alcohol_and_cancer_buykx_et_al.pdf

[B11] Cheeta S. Halil A. Kenny M. Sheehan E. Zamyadi R. Williams A. L. Webb L. 2018 Does perception of drug-related harm change with age? A cross-sectional online survey of young and older people *BMJ Open* 8 e021109 doi:10.1136/bmjopen-2017-021109 10.1136/bmjopen-2017-021109 PMC6231571 30401725

[B12] Choi Y.-J. Myung S.-K. Lee J.-H. 2018 Light alcohol drinking and risk of cancer: A meta-analysis of cohort studies *Cancer Research and Treatment* 50 474 487 doi:10.4143/crt.2017.094 28546524 10.4143/crt.2017.094 PMC5912140

[B13] Dixon H. G. Pratt I. S. Scully M. L. Miller J. R. Patterson C. Hood R. Slevin T. J. 2015 Using a mass media campaign to raise women’s awareness of the link between alcohol and cancer: Cross-sectional pre-intervention and post-intervention evaluation surveys *BMJ Open* 5 e006511 doi:10.1136/bmjopen-2014-006511 10.1136/bmjopen-2014-006511 PMC4360807 25762231

[B14] Éduc’alcool 2019 *Position statement: Warning labels on alcohol bottles* Montreal, Quebec Retrieved from http://educalcool.qc.ca/en/officialpositions/labels-on-bottles/#.XS4mJTY1u3A

[B15] Global Burden of Disease 2016 Alcohol Collaborators 2018 Alcohol use and burden for 195 countries and territories, 1990-2016: A systematic analysis for the global burden of disease study 2016 *The Lancet* 392 1015 1035 doi:10.1016/S0140-6736(18)31310-2 10.1016/S0140-6736(18)31310-2 PMC6148333 30146330

[B16] Global Burden of Disease 2017 Causes of Death Collaborators 2018 Global, regional, and national age-sex-specific mortality for 282 causes of death in 195 countries and territories, 1980-2017: A systematic analysis for the global burden of disease study 2017 *The Lancet* 392 1736 1788 doi:S0140-6736(18)32203-7 10.1016/S0140-6736(18)32203-7 PMC6227606 30496103

[B17] Global Burden of Disease Cancer Collaboration 2017 Global, regional, and national cancer incidence, mortality, years of life lost, years lived with disability, and disability-adjusted life-years for 32 cancer groups, 1990 to 2015: A systematic analysis for the global burden of disease study *JAMA Oncology* 3 524 548 doi:10.1001/jamaoncol.2016.5688 27918777 10.1001/jamaoncol.2016.5688 PMC6103527

[B18] Government of Northwest Territories 2017 *Northwest Territories Liquor Commission 2016-2017 63rd annual report* Hay River, Northwest Territories: Northwest Territories Finance Retrieved from http://library.assembly.gov.nt.ca/2017/F/a373811_td_451-182_Northwest_Territories_Liquor_Commission_63rd_annual_report.pdf

[B19] Government of Yukon 2017 *Yukon liquor corporation annual report April 1, 2016 to March 31, 2017* Whitehorse, Yukon Yukon Liquor Corporation Retrieved from http://www.ylc.yk.ca/pdf/YLC_annual_report_2016_17_WEB.pdf

[B20] Greenfield T. 1997 Warning labels: Evidence on harm reduction from long-term American surveys Plant M. Single E. Stockwell T. *Alcohol: Minimizing the harm* 105 125 London, England Free Association Books

[B21] Greenfield T. K. Graves K. L. Kaskutas L. A 1999 Long-term effects of alcohol warning labels: Findings from a comparison of the United States and Ontario, Canada *Psychology and Marketing* 16 261 282 doi:10.1002/(SICI)1520-6793(199905)16:3<261:: AID-MAR5>3.0.CO;2-Z

[B22] Hammond D. 2011 Health warning messages on tobacco products: A review *Tobacco Control* 20 327 337 doi:10.1136/tc.2010.037630 21606180 10.1136/tc.2010.037630

[B23] Heeb J. L. Gmel G. 2005 Measuring alcohol consumption: A comparison of graduated frequency, quantity frequency, and weekly recall diary methods in a general population survey *Addictive Behaviors* 30 403 413 doi:10.1016/j.addbeh.2004.04.022 15718058 10.1016/j.addbeh.2004.04.022

[B24] Hiilamo H. Crosbie E. Glantz S. A. 2014 The evolution of health warning labels on cigarette packs: The role of precedents, and tobacco industry strategies to block diffusion *Tobacco Control* 23 e2 doi:10.1136/tobaccocontrol-2012-050541 23092884 10.1136/tobaccocontrol-2012-050541 PMC3725195

[B25] Hobin E. Vallance K. Zuo F. Stockwell T. Rosella L. Simniceanu A. Hammond D. 2018 Testing the efficacy of alcohol labels with standard drink information and national drinking guidelines on consumers’ ability to estimate alcohol consumption *Alcohol and Alcoholism* 53 3 11 doi:10.1093/alcalc/agx052 29016708 10.1093/alcalc/agx052

[B26] Hydes T. J. Burton R. Inskip H. Bellis M. A. Sheron N. 2019 A comparison of gender-linked population cancer risks between alcohol and tobacco: How many cigarettes are there in a bottle of wine? *BMC Public Health* 19 316 doi:10.1186/s12889-019-6576-9 30917803 10.1186/s12889-019-6576-9 PMC6437970

[B27] International Agency for Research on Cancer 2008 *Methods for evaluating tobacco control policies. IARC handbooks of cancer prevention* 12 Lyon, France Author Retrieved from https://www.iarc.fr/wp-content/uploads/2018/07/Tobacco_vol12.pdf

[B28] International Agency for Research on Cancer 2010a *IARC monographs on the evaluation of carcinogenic risks to humans. Volume 96: Alcohol consumption and ethyl carbamate* Lyon, France Author Retrieved from https://monographs.iarc.fr/wp-content/uploads/2018/06/mono96.pdf PMC4781168 21735939

[B29] International Agency for Research on Cancer 2010b *Personal habits and indoor combustions. Volume 100 E: A review of human carcinogens**IARC monographs on the evaluation of carcinogenic risks to humans* Lyon, France Author Retrieved from https://monographs.iarc.fr/wp-content/uploads/2018/06/mono100E.pdf

[B30] Jongenelis M. I. Pratt I. S. Slevin T. Chikritzhs T. Liang W. Pettigrew S. 2018 The effect of chronic disease warning statements on alcohol-related health beliefs and consumption intentions among at-risk drinkers *Health Education Research* 33 351 360 doi:10.1093/her/cyy025 30085037 10.1093/her/cyy025

[B31] Kane L. 2018, January 3)Booze warning labels worked in U.S., says researcher after Yukon cautions pulled *The Canadian Press* Retrieved from https://nationalpost.com/pmn/news-pmn/canada-news-pmn/booze-warning-labels-worked-in-u-s-says-researcher-after-yukon-study-yanked

[B32] Kaskutas L. Greenfield T. K. 1992 First effects of warning labels on alcoholic beverage containers *Drug and Alcohol Dependence* 31 1 14 doi:10.1016/0376-8716(92)90002-T 1425211 10.1016/0376-8716(92)90002-t

[B33] Katikireddi S. V. Whitley E. Lewsey J. Gray L. Leyland A. H. 2017 Socioeconomic status as an effect modifier of alcohol consumption and harm: Analysis of linked cohort data *The Lancet Public Health* 2 e267 e276 doi:10.1016/S2468-2667(17)30078-6 28626829 10.1016/S2468-2667(17)30078-6 PMC5463030

[B34] LoConte N. K. Brewster A. M. Kaur J. S. Merrill J. K. Alberg A. J. 2018 Alcohol and cancer: A statement of the American Society of Clinical Oncology *Journal of Clinical Oncology* 36 83 93 doi:10.1200/JCO.2017.76.1155 29112463 10.1200/JCO.2017.76.1155

[B35] Manthey J. Shield K. D. Rylett M. Hasan O. S. M. Probst C. Rehm J. 2019 Global alcohol exposure between 1990 and 2017 and forecasts until 2030: A modelling study *The Lancet* 393 2493 2502 doi:S0140-6736(18)32744-2 10.1016/S0140-6736(18)32744-2 31076174

[B36] Martin N. Buykx P. Shevills C. Sullivan C. Clark L. Newbury-Birch D. 2018 Population level effects of a mass media alcohol and breast cancer campaign: A cross-sectional pre-intervention and post-intervention evaluation *Alcohol and Alcoholism* 53 31 38 doi:10.1093/alcalc/agx071 29155922 10.1093/alcalc/agx071

[B37] Martin-Moreno J. M. Harris M. E. Breda J. Møller L. Alfonso-Sanchez J. L. Gorgojo L. 2013 Enhanced labelling on alcoholic drinks: Reviewing the evidence to guide alcohol policy *European Journal of Public Health* 23 1082 1087 doi:10.1093/eurpub/ckt046 23657783 10.1093/eurpub/ckt046

[B38] Miller E. R. Ramsey I. J. Baratiny G. Y. Olver I. N. 2016 Message on a bottle: Are alcohol warning labels about cancer appropriate? *BMC Public Health* 16 139 doi:10.1186/s12889-016-2812-8 26864239 10.1186/s12889-016-2812-8 PMC4750299

[B39] Noar S. M. Francis D. B. Bridges C. Sontag J. M. Brewer N. T. Ribisl K. M. 2017 Effects of strengthening cigarette pack warnings on attention and message processing: A systematic review *Journalism & Mass Communication Quarterly* 94 416 442 doi:10.1177/1077699016674188 29975497 10.1177/1077699016674188 PMC5483339

[B40] Pettigrew S. Jongenelis M. Chikritzhs T. Slevin T. Pratt I. S. Glance D. Liang W. 2014 Developing cancer warning statements for alcoholic beverages *BMC Public Health* 14 786 doi:10.1186/1471-2458-14-786 25087010 10.1186/1471-2458-14-786 PMC4133604

[B41] Pettigrew S. Jongenelis M. I. Glance D. Chikritzhs T. Pratt I. S. Slevin T. Wakefield M. 2016 The effect of cancer warning statements on alcohol consumption intentions *Health Education Research* 31 60 69 doi:10.1093/her/cyv067 26787351 10.1093/her/cyv067 PMC4883036

[B42] Poirier A. E. Ruan Y. Volesky K. D. King W. D. O’Sullivan D. E. Gogna P. ComPARe Study Team 2019 The current and future burden of cancer attributable to modifiable risk factors in Canada: Summary of results *Preventive Medicine* 122 140 147 doi:10.1016/j.ypmed.2019.04.007 31078167 10.1016/j.ypmed.2019.04.007

[B43] Praud D. Rota M. Rehm J. Shield K. Zato ski W. Hashibe M. Boffetta P. 2016 Cancer incidence and mortality attributable to alcohol consumption *International Journal of Cancer* 138 1380 1387 doi:10.1002/ijc.29890 26455822 10.1002/ijc.29890

[B44] Probst C. Roerecke M. Behrendt S. Rehm J. 2014 Socioeconomic differences in alcohol-attributable mortality compared with all-cause mortality: A systematic review and meta-analysis *International Journal of Epidemiology* 43 1314 1327 doi:10.1093/ije/dyu043 24618188 10.1093/ije/dyu043 PMC4258771

[B45] Public Health Ontario 2017 *Alcohol: Awareness of alcohol-related health risks and support for health and nutrition labels* Retrieved from https://www.publichealthontario.ca/-/media/documents/alcohol-health-risks-labels.pdf?la=en

[B46] Rosenberg G. Bauld L. Hooper L. Buykx P. Holmes J. Vohra J. 2018 New national alcohol guidelines in the UK: Public awareness, understanding and behavioural intentions *Journal of Public Health* 40 549 556 doi:10.1093/pubmed.fdx126 28977621 10.1093/pubmed/fdx126 PMC6166584

[B47] Scheideler J. K. Klein W. M. P. 2018 Awareness of the link between alcohol consumption and cancer across the world: A review *Cancer Epidemiology, Biomarkers & Prevention* 27 429 437 doi:10.1158/1055-9965.EPI-17-0645 10.1158/1055-9965.EPI-17-0645 29615419

[B48] Stafford L. D. Salmon J. 2017 Alcohol health warnings can influence the speed of consumption *Journal of Public Health* 25 147 154 doi:10.1007/s10389-016-0770-3 28357194 10.1007/s10389-016-0770-3 PMC5350209

[B49] Strahan E. J. White K. Fong G. T. Fabrigar L. R. Zanna M. P. Cameron R. 2002 Enhancing the effectiveness of tobacco package warning labels: A social psychological perspective *Tobacco Control* 11 183 190 doi:10.1136/tc.11.3.183 12198266 10.1136/tc.11.3.183 PMC1759023

[B50] The Lancet 2018 Changing the conversation to make drug use safer [Editorial] *The Lancet* 391 doi:10.1016/S0140-6736(18)31075-4 10.1016/S0140-6736(18)31075-4 29864007

[B51] Vallance K. Romanovska I. Stockwell T. Hammond D. Rosella L. Hobin E. 2018 “We have a right to know”: Exploring consumer opinions on content, design and acceptability of enhanced alcohol labels *Alcohol and Alcoholism* 53 20 25 doi:10.1093/alcalc/agx068 29016716 10.1093/alcalc/agx068

[B52] Vallance K. Stockwell T. Hammond D. Shokar S. Schoueri-Mychasiw N. Greenfield T. Hobin E. 2020 Testing the effectiveness of enhanced alcohol warning labels and modifications resulting from alcohol industry interference in Yukon, Canada: Protocol for a quasi-experimental study *JMIR Research Protocols* 9 1 e16320 doi:10.2196/16320 31922493 10.2196/16320 PMC6996737

[B53] Weerasinghe A. Schoueri-Mychasiw N. Vallance K. Stockwell T. Hammond D. McGavock J. Hobin E. 2020 Improving knowledge that alcohol can cause cancer is associated with consumer support for alcohol policies: Findings from a real-world alcohol labelling study *International Journal of Environmental Research and Public Health* 17 398 doi:10.3390/ijerph17020398 10.3390/ijerph17020398 PMC7014334 31936173

[B54] Weiss B. D. Mays M. Z. Martz W. Castro K. M. DeWalt D. A. Pignone M. P. Hale F. A. 2005 Quick assessment of literacy in primary care: The newest vital sign *Annals of Family Medicine* 3 514 522 doi:10.1370/afm.405 16338915 10.1370/afm.405 PMC1466931

[B55] Wettlaufer A. 2018 Can a label help me drink in moderation? A review of the evidence on standard drink labelling *Substance Use & Misuse* 53 585 595 doi:10.1080/10826084.2017.1349798 28937874 10.1080/10826084.2017.1349798

[B56] Wigg S. Stafford L. D. 2016 Health warnings on alcoholic beverages: Perceptions of the health risks and intentions towards alcohol consumption *PLoS One* 11 4 e0153027 doi:10.1371/journal.pone.0153027 27105210 10.1371/journal.pone.0153027 PMC4841515

[B57] Wiseman K. P. Klein W. M. P. 2019 Evaluating correlates of awareness of the association between drinking too much and cancer risk in the United States. *Cancer Epidemiology* *Biomarkers & Prevention* 28 1195 1201 doi:10.1158/1055-9965.EPI-18-1010 10.1158/1055-9965.EPI-18-1010 PMC6606368 31043419

[B58] World Cancer Research Fund/American Institute for Cancer Research 2007 *Food, nutrition, physical activity, and the prevention of cancer: A global perspective* Washington, DC AICR 10.1017/S002966510800712X 18452640

[B59] World Health Organization 2010 *Global strategy to reduce the harmful use of alcohol* Geneva, Switzerland Author Retrieved from https://www.who.int/substance_abuse/publications/global_strategy_reduce_harmful_use_alcohol/en/

[B60] World Health Organization 2017 *Alcohol labelling: A discussion document on policy options* Copenhagen, Denmark WHO Regional Office for Europe Retrieved from http://www.euro.who.int/__data/assets/pdf_file/0006/343806/WH07_Alcohol_Labelling_full_v3.pdf?ua=1

[B61] World Health Organization 2018 *Global health observatory data repository (European Region): Health warning labels on alcohol containers by country* Retrieved from http://apps.who.int/gho/data/node.main-euro.A1193?lang=en&showonly=GISAHFIGURE

